# An individualized decision aid for physicians and patients for total knee replacement in osteoarthritis (Value-based TKR study): study protocol for a multi-center, stepped wedge, cluster randomized controlled trial

**DOI:** 10.1186/s12891-021-04546-5

**Published:** 2021-09-12

**Authors:** Toni Lange, Stefanie Deckert, Franziska Beyer, Waldemar Hahn, Natascha Einhart, Martin Roessler, Martin Sedlmayr, Jochen Schmitt, Jörg Lützner

**Affiliations:** 1 Center for Evidence-based Healthcare, University Hospital Carl Gustav Carus and Faculty of Medicine Carl Gustav Carus, Technische Universität Dresden, Dresden, Germany; 2 University Center of Orthopedics, Trauma and Plastic Surgery, University Hospital Carl Gustav Carus Dresden, Technische Universität Dresden, Fetscherstr. 74, 01307 Dresden, Germany; 3 Institute for Medical Informatics and Biometry, Faculty of Medicine Carl Gustav Carus, Technische Universität Dresden, Dresden, Germany

**Keywords:** Shared decision making, Total knee arthroplasty, Decision quality, Patient-reported outcome measures

## Abstract

**Background:**

Total knee replacement (TKR) is one of the most commonly performed routine procedures in the world. Prognostic studies indicate that the number of TKR will further increase constituting growing burden on healthcare systems. There is also substantial regional heterogeneity in TKR rates within and between countries. Despite the known therapeutic effects, a subset of patients undergoing TKR does not benefit from the procedure as intended. To improve the appropriateness of TKR indication, the EKIT initiative (“evidence and consensus based indication critera for total arthroplasty”) developed a clinical guideline for Germany on the indication of TKR. This guideline is the basis for a digital medical decision aid (EKIT tool) to facilitate shared decision making (SDM) in order to improve decision quality for elective surgery. The aim of this cluster randomized trial is to investigate the effectiveness of the EKIT tool on decision quality.

**Methods:**

The Value-based TKR study is a prospective pragmatic multi-center, stepped wedge, cluster randomized controlled trial (SW-RCT). The EKIT tool provides (1) a systematic presentation of individual patient and disease-specific information (symptoms, expectations), (2) the fulfillment of the indication criteria and (3) health information about safety and effectiveness of TKR. All study sites will follow routine care as control clusters until the start of the intervention. In total, there will be 10 clusters (study sites) and 6 sequential steps over 16 month, with clusters receiving the intervention with a minimum 2 months of standard routine care. The primary outcome is patients’ decision quality measured with the Decision Quality Instrument (DQI)-Knee Osteoarthritis questionnaire. Furthermore, we will collect information on global patient satisfaction, patient reported outcome measures and the fulfilment of the individual expectations 12 months after SDM. The power calculation yielded an estimated power of 89% using robust Poisson regression under the following assumptions: 10 study sites with a total of N=1,080 patients (including a dropout rate of 11%), a 10% increase in decision quality due to the use of the EKIT tool, and a significance level of 5%.

**Discussion:**

There is a high potential for transferring the intervention into routine practice if the evaluation is positive.

**Trial registration:**

ClinicalTrials.gov: NCT04837053. Registered on 08/04/2021.

## Background

Knee osteoarthritis (OA), the most common joint disease in the world, is estimated to affect more than 650 million people [1, 2]. It is one of the most common causes of pain and disability in older people [3]. There is currently no causal drug therapy for knee OA [4]. However, for end-stage osteoarthritis, elective joint replacement surgery is a cost-effective treatment option [5, 6]. Total joint replacement (TJR) is one of the most commonly performed routine procedures in the world [7]. There is, however, substantial regional heterogeneity in total knee replacements (TKR) rates within and between countries [6, 8–10]. Based on the demographic change, a further increase in the number of TKR can be assumed [9], which might additionally further increase the heterogeneity of care. Prognoses impressively show the future significant impact on the health care system [11–18]. This is reflected in expected cost increases, but also in increased demand for resources from providers.

Despite the known therapeutic effects, such as pain reduction [6, 19], improvement of function [19–21] and health related quality of life [21–23], a subset of patients undergoing TKR does not benefit from the procedure as intended: (1) the proportion of patients with (residual) pain in the long-term course has been reported between 10 and 34 % [24], (2) residual symptoms and functional limitations between 33 and 54 % [25] and (3) the proportion of patients which are not satisfied has been reported recently still between 12 and 15 % [24, 26]. It was shown that patients whose expectations were fulfilled were also more satisfied with the outcome than those whose expectations were not or only insufficiently fulfilled [27–29]. It has been demonstrated that expectations were modifiable [30]. Consequently, the knowledge of the patient's expectations and treatment goals is necessary for (1) predicting treatment outcomes and, subsequently, for (2) patient-centered indications for elective surgery. Furthermore, these expectations on the surgery should be considered in (3) shared decision making (SDM) in order to address any unrealistic treatment goal and to adjust the therapy or the expectation accordingly.

Standardized assessment of patient and physician expectations related to elective surgery led to more effective consideration of patient goals in the decision making process [31]. Another positive aspect of the standardized collection of patient expectations and treatment goals is the possible individualization of treatment options according to the patient's preferences and needs.

Internationally, the review of Gademan et al [32] summarized the current state of science for the indication for TKR. This overview identified six guidelines for TKR. However, consideration of patient expectations was not included in the recommendations. Therefore, the EKIT initiative (“evidence and consensus based indication criteria for total arthroplasty”) developed a clinical guideline for Germany on the indication of TKR considering treatment goals and the associated probability of achievement [33]. This formed the basis for the idea developing a digital medical decision aid (EKIT tool) that facilitates SDM incorporating patient`s symptoms, expectations and the guideline for physicians aiming to improve decision quality. Overall, medical decision aids can be used as tool for structured SDM [34] and facilitate the communication in the decision-making process [35]. Ideally, the medical decision aids will help to increase the quality of decision-making for both physicians and patients. However, to the best of our knowledge, there is a lack of studies on SDM tools that interact between both physician and patient [36].

Therefore, the aim of this cluster randomized trial is to investigate the effectiveness of the EKIT tool to improve decision quality of the patients. The tool will provide physicians with information about patient-specific and disease-specific factors (predictors) as well as treatment goals and patient preferences (desired outcome) for indication and subsequently visualizes health information for a guided SDM.

## Methods / Design

The SPIRIT (Standard Protocol Items for Randomized Trials) [37], the SPIRIT extension for Patient-Reported Outcomes [38] and SUNDAE (Standards for Universal Reporting of Decision Aid Evaluations) recommendations [39] were used during the whole process of protocol development. Additionally, we followed the extension for stepped wedge cluster randomized trials of the CONSORT (Consolidated Standards of Reporting Trials) statement [40] as supplement for reporting the study design. This trial was registered on ClinicalTrials.gov (NCT04837053, 08/04/2021).

### Study design

**Fig. 1:**
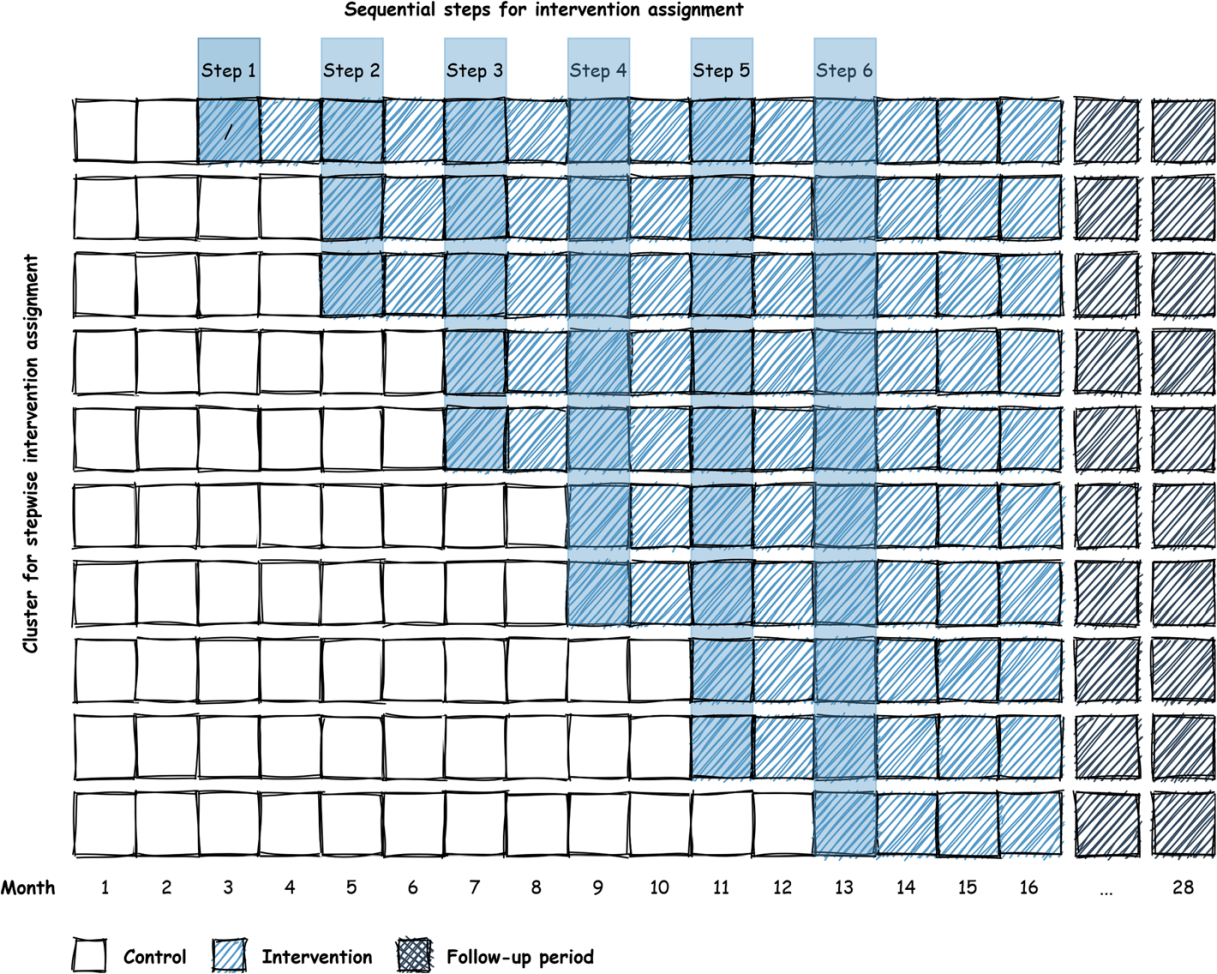
The stepped wedge design as applied in Value-based TKR

The Value-based TKR study is a prospective pragmatic multi-center, stepped wedge, cluster randomized controlled trial (SW-RCT). Each cluster will be randomly assigned to interventions on a staged schedule. In total, there will be 10 clusters (study sites) which will change from control (routine care) to the intervention (EKIT tool) in a predefined randomly allocated sequence. Each cluster will have a minimum 2 months of routine care before changing to the intervention (Fig. 1). At the end of the study, all clusters will be assigned to the intervention with a minimum of 4 months. The total duration of study will be 16 months.

The rationale behind the design selection was (1) to avoid influencing routine care with knowledge of the intervention, (2) to deploy the digital intervention at the cluster level, as the study intervention should be integrated into routine care, (3) the intention to leave the intervention in place at the end of the study and (4) facilitates cluster recruitment as it enhances the acceptability.

### Study aims

The purpose of this study is (1) to compare the effectiveness of the EKIT tool versus routine care in terms of decision quality and satisfaction with the SDM. Furthermore, we aim to (2) investigate with a 12-month follow-up the impact of the EKIT tool on treatment choices, patient reported outcomes measures (PROM) and satisfaction with treatment outcome. (3) We aim to investigate associations that influence the decision quality on patient and intervention level. Specific study hypotheses are:

- *Hypothesis 1*: Patients receiving the intervention have higher decision quality than patients receiving routine care.
- *Hypothesis 2*.1: Patients report higher satisfaction with the SDM when the EKIT tool was used.
- *Hypothesis 2*.2: Physicians report higher satisfaction with the SDM when the EKIT tool was used.
- *Hypothesis 3.1:* After 12 months, patients are more satisfied with their chosen therapy if the EKIT tool was used.
- *Hypothesis 3.2:* After 12 months, patients are more satisfied with the treatment decision made if the EKIT tool was used.
- *Hypothesis 3.3:* After 12 months, individual patient expectations are more likely fulfilled if the EKIT tool was used.

### Participants, interventions, and outcomes

#### Study setting

The study will take place in ten surgical centers (study sites) in Germany. During routine care, patients are referred to a surgical center by their outpatient primary care physician or orthopedic specialist for a TKR. Study sites will be chosen from different level of care and infrastructural regions in Germany.

#### Eligibility criteria

##### Cluster inclusion criteria for SW-RCT

Study sites will be included if there is a high probability that the needed sample size may be recruited. Therefore, the volume of at least 200 primary TKR’s per year was required.

##### Patient inclusion criteria for SW-RCT

The target population includes all patients with knee osteoarthritis which are referred for TKR by the treating physician. Eligibility criteria are: (1) patient with knee osteoarthritis who is a candidate for total knee replacement, (2) capacity to consent, (3) understanding of the German language (written and oral), (4) age of 18 years or older, and (5) informed consent. Patients with already performed knee replacement ipsilateral will be excluded from the study.

#### Interventions

##### Intervention cluster - EKIT tool

**Fig. 2:**
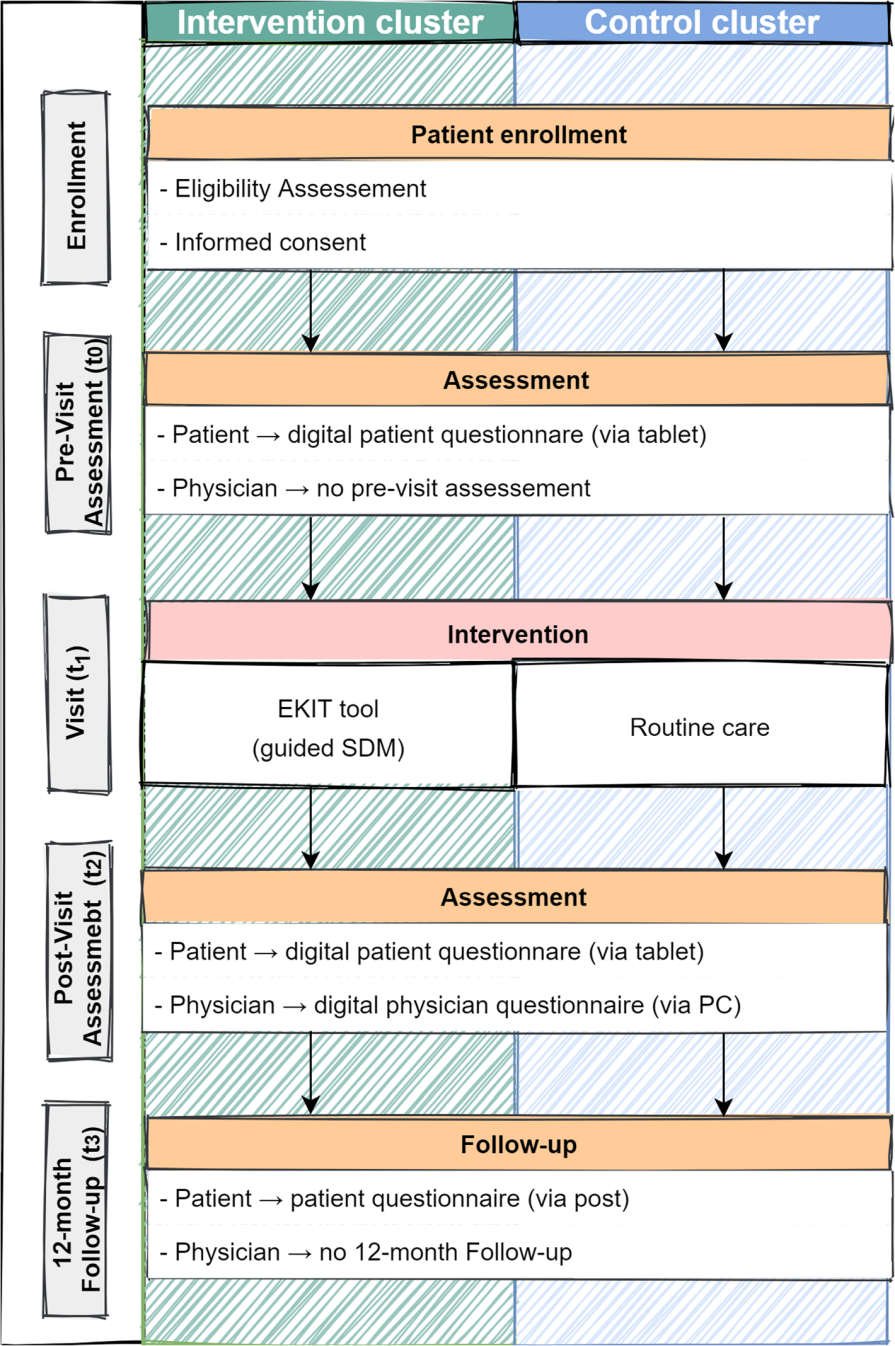
Flow of study intervention and assessment

The intervention (EKIT tool) consists of three parts (Fig. 2). (1) To guide the SDM process and to support the consultation, the EKIT tool provides a systematic presentation of individual patient and disease-specific information and (2) it visualizes the fulfillment of the indication criteria in Germany. (3) To empower the patient, the EKIT tool additionally presents health information about TKR adopted from the health information developed by the German independent Institute for Quality and Efficiency in Health Care (Institut für Qualität und Wirtschaftlichkeit im Gesundheitswesen, IQWIG).

1. Individual patient information

In addition to the visualization of clinically assessed parameters (e.g. the individual Oxford Knee Score (OKS) in comparison to the OKS distribution before TKR), the EKIT tool presents the expectations and the associated probabilities of fulfillment as estimated by the physician. This combines patient`s and surgeon`s perspective on the TKR.

(2) Indication criteria for TKR in Germany

The German guideline on TKR indication is available on the website of the Association of the Scientific Medical Societies in Germany (AWMF, https://www.awmf.org/leitlinien/detail/ll/033-052.html) and furthermore published in short form for the German orthopedic community [41] and the conceptual framework with the resulting criteria is also available in English [33]. The EKIT tool visualizes the degree of fulfillment of the guideline recommendations for the indication criteria knee TKR. The following five core indication criteria were agreed within the EKIT panel: (1) intermittent (several times per week) or constant knee pain for at least 3–6 months; (2) radiological confirmation of structural knee damage (osteoarthritis, osteonecrosis); (3) inadequate response to conservative treatment, including pharmacological and non-pharmacological treatment for at least 3–6 months; (4) adverse impact of knee disease on patient’s quality of life for at least 3–6 months; (5) patient-reported suffering/impairment due to knee disease.

(3) Health information about TKR

The health information adapted from the IQWIG describes the frequency of adverse events, return to activities and the potential of TKR to reduce discomfort and pain.

##### Control cluster

All study sites will follow routine care as control clusters until the start of the intervention. However, there are additional study –specific data collection processes besides routine care. This deviation from the routine care for participating study sites is necessary to evaluate the effectiveness of the intervention. We have made every effort to keep potential bias to a minimum. The additional data collection includes sociodemographic data, PROM, treatment goals, clinician-based outcomes, information about decision-making, patient participation, and previous therapies (Table 1).

**Table 1:** Summary of outcomes, measurement instruments and time of measurement

Outcome Domain	Measurement instrument	Source	Assessor perspective		Study period						Data type
			Patient	Clinician	Enrolment	Pre-Visit (t_**0**_)	Visit (t_**1**_)	Post-Visit (t_**2**_)	Follow-up (t_**3**_)	Close-out	
**Enrolment**											
Eligibility screen					x						**-**
Informed consent					x						**-**
**Intervention**											
Decision aid							x				**-**
Routine care							x				**-**
**Assessments**											
Sociodemographic data											
Sociodemographic	Sociodemographic questionnaire	Self-made				x					-
PROM											
Pain & function	Oxford Knee Score (OKS)	[42]	x			x			x		con (0-48)
Generic health related quality of life	EQ-5D-5l	[43]	x			x			x		con (0-1)
Subjective suffering, health related quality of life, pain	Numeric rating scale (0-10)	Self-made	x			x			x		con (0-10)
Pain	Patient survey	Self-made	x			x			x		cat
Muscoloscleral complaints	Musculoskeletale morbidities	Self-made	x			x			x		cat
Global treatment outcome	5-point Likert-scale	[44]	x						x		cat
Disease-related Symptoms, complaints disabilities	3-point Likert-scale	Self-made	x			x			x		cat
Treatment goals											
Expectations	3-point Likert-scale	Self-made, [45]	x			x					cat
Fullfilment of expectation	5-point Likert-scale	Self-made	x						x		cat
Estimation of the probability of fulfillment	5-point Likert-scale	Self-made, [45]		x		x					cat
CBO											
Range of motion	Angular measurement			x				x^b^			con
Stability	Joint clearance : Mediolateral and anteroposterior			x				x^b^			cat
BMI	Weight and length measurement		x					x^b^			con
Morbidity	Anamnesis			x				x^b^			cat
Contraindication	Anamnesis			x				x^b^			cat
Degree of Osteoarthritis	Kallgren & Lawrence			x				x^b^			cat
Joint space narrowing	X-ray			x				x^b^			cat
Leg axis position	X-ray			x				x^b^			cat
Decision making and patient participation											
Participation in therapy decision	PEF–FB-9	[46]	x					x			con (0-100)
Preference for involvement in decision-making	Control Preferences Scale (CPS)	[47]	x			x					cat
Decision quality	DQI-Knee Osteoarthritis^a^	[48]	x					x	x		cat
	Decision Process Score of the DQI-Knee Osteoarthritis^a^	[48]	x					x			con (0-100)
	4-point Likert Scare	Selfmade				x		x			cat
Decision regret	Decision Regret Scale (DRS)	[49]	x						x		con (0-100)
Satisfaction with consultation (physician)	5-point Likert-scale	Self-made	x					x			cat
Satisfaction with consultation (patient)	5-point Likert-scale	Self-made		x				x			cat
Agreed medical treatment decision	NA	Self-made		x				x			cat
Adherence to clinical guideline	Fulfillment of indication criteria	Self-made		x				x	x		cat
Previous therapy											
Conservative therapy	Patient survey	Self-made	x			x			x		-
Operative therapy	Patient survey	Self-made	x			x					-

^a^ Modified version of the DQI- Knie; PROM: Patient reported outcome measure; CBO: Clinician-based outcome; Categorial (cat); Continuous (con) ^b^ in the intervention group during counseling

#### Outcomes

##### Primary outcome

The primary outcome for this trial is the patient’s decision quality after the patient-physician consultation. The binary outcome of the informed patient-centered decision will be measured with the Decision Quality Instrument (DQI)-Knee Osteoarthritis [48] questionnaire and reflects the extent that patients are informed (at least three out of five knowledge questions are correct, ≥ 60% in the knowledge score) and receive their preferred treatment (conservative vs. TKR). In detail, the proportion of right answers of the five item knowledge questionnaire are scored on a scale ranging from 0% to 100%. To obtain concordance between preferences and treatment received, the patient is asked about the preferred treatment immediately after the consultation (t_2_) and the treatment received is reported within the follow-up 12 months after the consultation (t_3_). Finally, an informed patient-centered decision is present if the individual knowledge score is ≥ 60% and the preferred and received treatment are concordant. The primary analysis examines differences between intervention and control regarding the probability of informed patient-centered decisions.

##### Secondary outcomes

Patient and physician satisfaction measured on a 5-point Likert-scale with the SDM will be analyzed regarding intervention effects. Furthermore, we will collect the following secondary outcomes 12 month after SDM: patients satisfaction with (1) their chosen therapy measured by the global treatment outcome (GTO) [44] and (2) treatment decision measured by the Decision Regret Scale (DRS) [49] to determine if there is exploratory evidence of differences between intervention and control. We will further investigate the fulfilment of the individual expectation between the groups measured on a 5-point Likert-scale, 12 months after SDM. Details on secondary outcome parameters, measurement instruments and time frames are listed in Table 1.

##### Participant timeline

The participant timeline contains the enrollment, two digital assessments (t_0_, t_2_) and the follow-up (t_3_) 12 months after consultation (Fig. 2). The data collected at the various time points are shown in Table 1.

##### Enrollment and pre-Visit assessment(t0)

After screening the patients for inclusion and exclusion criteria within each study site, eligible patients will be informed about the study and asked for participation. Patients willing to participate will sign informed consent and will fill in a digital questionnaire (Table 1) prior to the patient-physician consultation.

##### Intervention (t1)

The study physician will perform clinical examination as usual. Depending on whether the study center is already in the intervention phase, (1) SDM will be performed as usual in routine care or (2) the SDM process will be guided using the EKIT tool.

1. In the control phase, the study physician will not have access to the additional previously collected patient data. He/she will perform SDM as usual. Information about the chosen therapy will be given as usual.
2. In the intervention phase, the SDM process is guided by the EKIT tool. If TKR is indicated, the patient will receive standardized information about the advantages and disadvantages of the TKR surgery based on IQWIG health information.

##### Post-Visit assessment (t2)

During the control phase and the intervention phase, both the physician and the patient will fill in a digital questionnaire separately. The physician will be asked about her/his satisfaction with the SDM process and will justify the therapy decision on a standardized questionnaire. The patient will be asked about her/his satisfaction with the SDM process and will answer five standard questions about TKR.

##### Follow-Up (t3)

The follow-up will take place 12 months after SDM. Study staff will contact all patients by mail to fill in a questionnaire including current global health status, disease-specific complaints, degree of fulfillment of preoperative expectations, patient satisfaction with the treatment outcome and satisfaction with decision-making and global treatment outcome.

#### Statistical power

The power calculation was based on the primary study outcome (decision quality). A rather conservative scenario to estimate the expected power of the confirmatory analysis was chosen. The calculation was performed for a stepped wedge cluster randomized trials design with a recruitment period of 16 months. Based on a previous study on the effectiveness of a decision aid for patients with osteoarthritis [50], the baseline probability of ‘good decision quality’ measured by DQI-Knee Osteoarthritis [48] was assumed to be 44.5%. Furthermore, we assumed a 10 % increase in decision quality by the implementation of the EKIT tool as decision aid.

We expected 10 participating study sites with an average number of 108 enrolled patients per site over the entire time period (N total = 1,080), accounting for an estimated dropout rate of 11%. The intervention (EKIT tool) was assumed to be implemented sequentially as described in Fig. 1. The modeling of possible correlations within the clusters was performed as part of the data generation process using a logistic multilevel model with a standard deviation of the random intercept of 0.01. Based on these assumptions and a significance level of 5%, the assumed intervention effect on decision quality can be estimated using robust Poisson regression with a power of 89%. The power calculation was performed with the statistical software R [51].

#### Recruitment

Each study site was checked for (1) having the necessary number of eligible patients and (2) necessary infrastructure to perform the study. Each study site will be given target numbers for patients to be enrolled in the study per study stage (control, intervention phase). Study sites will competitively recruit up to three consecutive patients per week (the first eligible patients). Achievement of the target numbers will be checked on a monthly basis by the study coordinator. In the case that overall recruitment is 20% lower than planned, the rollout of the interventions will be delayed for all study sites.

### Assignment of interventions

#### Allocation

To minimize the potential bias associated with the fact that the study PI (who already knows the intervention) is also part of a study site, the PI will not enroll patients into the control group, only other study physicians at his site. Randomization of the ten study sites into six sequential steps will be based on computer generated random numbers by the study statistician. The study management will not have the opportunity to influence the randomization process. Consequently, each cluster will initialize the intervention according to the randomly allocated staggered implementation schedule. Study sites gave their consent for the timing of the start of the intervention to be determined by randomization. The study team is responsible for coordinating the timing of study initiation based on the generated allocation sequence.

#### Blinding

Patients, physicians and study staff as well as the study statistician cannot be blinded to the assigned study arm. However, the patients will not receive any further explanation as to which study arm they were allocated to.

### Data collection, management, and analysis

#### Data collection methods and data management

Overall, the majority of data will be collected electronically, with the exception of the follow-up survey. Figure 2 summarizes the data collection process in this study. A web application platform for collection of the data was developed for this study. This web application includes the questionnaires in both phases and additionally the EKIT tool in the intervention phase. Each questionnaire is checked by the web application, ensuring valid answers for all required questions. All data protection issues are detailed in a separate data protection concept.

The follow-up questionnaire (t_3_) will be sent by mail 12 months after t_1_. After four weeks without a response, study staff will send a reminder for the follow-up survey (t_3_). Patients will receive no kinds of incentives or compensation for participation in this study.

#### Statistical methods

All analyses will be evaluated by intention-to-treat principles in terms of assignment to treatment and use a level of significance of 0.05. Study sites, which stop participation after the trial start date or will not implement intervention, will be included in the analysis. There are no interim-analyses planned for this study. We will use the statistical software R [51] for all analyses.

##### Confirmatory analysis

The confirmatory analysis of the primary outcome (decision quality, DQI-Knee Osteoarthritis) [48] is performed as complete case-analysis by estimating the effect of EKIT tool implementation on decision quality using robust Poisson regression for clustered data [52]. The use of this method allows direct estimation of the relative increase in the probability of good decision quality while adequately accounting for the data structure. Furthermore, a regression-based approach allows adjustment for other relevant covariates in the context of sensitivity analyses. Interim analyses were not planned in this study.

##### Sensitivity analysis

In the sensitivity analyses, we will impute missing values of the outcomes and covariates using MICE. Furthermore, we will compare the results of the primary analysis with the results for different subgroups to assess the stability of the results.

##### Explorative analysis

The statistical analysis of the secondary outcome parameters is exploratory-hypothesis generating. In addition to descriptive standard methods, multilevel models will be used to explore potential factors influencing the outcome parameters. Multiple Imputation by Chained Equations (MICE) will be used for the imputation of missing data in the explorative analysis. In addition, we plan to investigate the cohort with regard to epidemiological questions for influencing factors that have a predictive impact on outcomes 12 months after surgery. Furthermore, we will explore the adherence to the clinical guideline.

#### Process evaluation

The acceptance of the EKIT tool will be investigated via qualitative research methods as a form of process evaluation. Therefore, participating surgeons and patients will be interviewed to obtain information on the acceptance and usability of the EKIT tool. Furthermore, influencing factors and barriers to a broad implementation of the EKIT tool will be identified.

### Monitoring

This study does not integrate external staff for data monitoring procedures as the risk for patients or other involved personnel of this study is minimal. Internal data monitoring is coordinated by the study coordinator. For this purpose, random sets (n = 10) of de-identified patient information from each study site will be requested and checked.

To monitor the recruitment process, an internal study review board will meet every three months and inform the study sites about the recruitment process if necessary. In addition, study progress reports will be submitted quarterly to the funder (Innovation Fund of the Joint Federal Committee) independent of internal data monitoring.

### Ethics and dissemination

#### Protocol version

This study protocol obtained ethical approval by the Ethical Committee of the Faculty of Medicine of the TU Dresden (institutional review board) in August 2020 (EK-271062020).

#### Protocol amendments

All changes to the study protocol were documented and submitted to the institutional review board for information. All participating study centers will receive regular information about the recruitment process and updates of the study protocol. Furthermore, all major changes in the study protocol will be added on ClinicalTrials.gov

#### Study participant consent

##### Surgeon consent

The principal investigator (JL) corresponded with the study sites. In addition, all participating study stuff from each study site will attend a kickoff meeting and each study site will have a site initiation. Participating study sites provided written consent to participate in the study.

##### Patient consent

Eligible patients will be informed about the study and about the privacy protection concept prior to study participation and will sign an informed consent.

#### Confidentiality

Efforts to protect privacy were described in a separate data protection concept. The data protection concept includes the description of the consistent separate treatment of personal identification data and medical data as well as the data flows and transfer protocols, storage locations, security, access rights and behavior in the event of deletion requests.

#### Dissemination plan

Patient representatives from Deutsche Rheumaliga as well as representatives of statutory health insurances (AOK) are involved in the whole project which consists of the development of the EKIT-tool, this cluster RCT, an additional qualitative study on the feasibility of the EKIT tool and finally a workshop for the discussion of the results and planning of the further implementation of the EKIT tool. The study team will publish the results completely and independently of the project results as an open access publication in a leading peer-reviewed journal to reach a broad audience. We will report in accordance with the CONSORT 2010 statement and the extension for stepped wedge cluster randomized trials. Furthermore, we will disseminate the results by presenting the results at conferences for orthopaedic surgeons.

#### Availability of data and materials

The raw and analyzed datasets of this study are not publicly available as participants of this study did not agree for their data to be shared publicly. Aggregated study results will be available from corresponding author on reasonable request.

## Discussion

The intervention in this study aims to improve decision quality for or against knee replacement. Therefore, the primary outcome parameter was chosen accordingly. However, the intervention also aims to improve the transfer/integration of the German guideline recommendations for the indication of TKA into routine care and therefore, also to improve the quality of the indication.

### Strength and limitations

The generalizability of the study results is subject to several limitations. Patients not familiar with digital interventions may need a study staff member to assist during input into the digital data collection forms. This may limit the pragmatic implementation of the intervention and external validity of the study. The acceptance of the intervention by patients and physicians will be investigated as supporting research, but currently there is only limited knowledge about the acceptance of a digital intervention into routine care based on the previous feasibility studies. This may affect the recruiting phase.

We have developed a new decision tool to guide the shard decision process and assume to address all of the key elements provided by Riddle, Sando [36]: (1) situations diagnosis, (2) choice awareness, (3) option clarification, (4) discussion of harms and benefits, (5) deliberation of patient preferences and (6) making the decision. Furthermore, this study investigates four out of five core domains of the preliminary core domain set of outcomes for SDM interventions [53]. This includes the investigation of (1) knowledge, (2) concordance between treatment options and preferences, (3) satisfaction with the decision making-process (4) adherence to the chosen option. The assessment of confidence in the decision made is not integrated in this study.

### Methodological considerations

Compared to conventional parallel cluster randomized controlled trial (cRCT), the choice of the stepped wedges design has a higher risk of bias due to time effects from the staggered nature of the roll-out [54] e.g. if there are secular trends associated with the study outcome [55]. Furthermore, the risk for within-cluster contamination bias is higher than in conventional parallel cRCT [54]. However, we expect these effects to be minimal in this study because there is no knowledge about secular effects on the outcomes (e.g. decision quality and satisfaction) and, if the control phase is successfully implemented, the transition time and expense is minimal.

The power calculation was based on Monte Carlo simulation since analytical solutions for stepped wedged designs drawing on nonlinear statistical models have not yet been established [56]. The systematic review of Eichner, Groenwold [57] shows concerns about reaching the sample size with the stepped wedge design. However, we assume that the advantages outweigh these considerations. To counteract the threat of not reaching a sufficiently large sample size, we made careful arrangements with the study sites before the start of the study.

### Future research and perspective

In case of a successful evaluation, the web application will be further developed to allow the collection of follow-up data via web-based survey and return summary of individual's own postoperative results compared to the overall cohort. There is a high potential for transferring the intervention into routine practice if the evaluation is positive, as the intervention is complex but also pragmatic.

## Data Availability

Not applicable.

## Abbreviations

AWMF: Arbeitsgemeinschaft der Wissenschaftlichen Medizinischen Fachgesellschaften
Cat: Categorial
CBO: Clinician-based outcome
cRCT: Cluster randomized controlled trial
CONSORT: Consolidated Standards of Reporting Trials
Con: Continuous
CPS: Control Preferences Scale
DQI: Decision quality index
DQI: Decision Quality Instrument
DRS: Decision Regret Scale
EKIT: Evidence and consensus based indication criteria for total arthroplasty
GTO: Global treatment outcome
IQWIG: Institut für Qualität und Wirtschaftlichkeit im Gesundheitswesen
OA: Knee osteoarthritis
MICE: Multiple Imputation by Chained Equations
OKS: Oxford Knee Score
PEF–FB-9: Participation in therapy decision
PROM: patient reported outcomes measures
SDM: Shared decision making
SPIRIT: Standard Protocol Items for Randomized Trials
SUNDAE: Standards for Universal Reporting of Decision Aid Evaluations
SW-RCT: stepped wedge, cluster randomized controlled trial
TJR: Total joint replacement
TKR: Total knee replacements
